# Molecular Pathology of Rare Bleeding Disorders (RBDs) in India: A Systematic Review

**DOI:** 10.1371/journal.pone.0108683

**Published:** 2014-10-02

**Authors:** Bipin P. Kulkarni, Sona B. Nair, Manasi Vijapurkar, Leenam Mota, Sharda Shanbhag, Shehnaz Ali, Shrimati D. Shetty, Kanjaksha Ghosh

**Affiliations:** National Institute of Immunohaematology (I.C.M.R.), MS Building, KEM Hospital campus, Parel, Mumbai, India; Odense University hospital, Denmark

## Abstract

**Background:**

Though rare in occurrence, patients with rare bleeding disorders (RBDs) are highly heterogeneous and may manifest with severe bleeding diathesis. Due to the high rate of consanguinity in many caste groups, these autosomal recessive bleeding disorders which are of rare occurrence in populations across the world, may not be as rare in India.

**Objectives:**

To comprehensively analyze the frequency and nature of mutations in Indian patients with RBDs.

**Methods:**

Pubmed search was used (www.pubmed.com) to explore the published literature from India on RBDs using the key words “rare bleeding disorders”, “mutations”, “India”, “fibrinogen”, “afibrinogenemia”, “factor II deficiency”, “prothrombin” “factor VII deficiency”, “factor V deficiency”, “factor X deficiency”, “factor XI deficiency”, “combined factor V and VIII deficiency”, “factor XIII deficiency”, “Bernard Soulier syndrome” and “Glanzmanns thrombasthenia” in different combinations. A total of 60 relevant articles could be retrieved. The distribution of mutations from India was compared with that of the world literature by referring to the Human Gene Mutation Database (HGMD) (www.hgmd.org).

**Results:**

Taken together, 181 mutations in 270 patients with different RBDs have been reported from India. Though the types of mutations reported from India and their percentage distribution with respect to the world data are largely similar, yet much higher percentage of small deletions, duplication mutations, insertions, indels were observed in this analysis. Besides the identification of novel mutations and polymorphisms, several common mutations have also been reported, which will allow to develop a strategy for mutation screening in Indian patients with RBDs.

**Conclusion:**

There is a need for a consortium of Institutions working on the molecular pathology of RBDs in India. This will facilitate a quicker and cheaper diagnosis of RBDs besides its utility in first trimester prenatal diagnosis of the affected families.

## Introduction

Amongst bleeding disorders, hemophilia A (FVIII deficiency), hemophilia B (FIX deficiency) and von Will brand disease (vWD) are most commonly occurring, whereas deficiencies of fibrinogen, prothrombin (FII), factor V (FV), combined factor V and VIII (FV+VIII), factor VII (FVII), factor X (FX), factor XI (FXI) and factor XIII (FXIII), along with platelet disorders, Glanzmanns Thrombasthenia (GT) and Bernard Soulier Syndrome (BSS) are rare worldwide, including India. Hemophilias are X chromosome linked disorders, whereas the inheritance pattern of the RBDs is generally autosomal recessive [1].

Patients with rare coagulation factor deficiencies may manifest with severe bleeding diathesis. Even with severe deficiencies of different coagulation factors, bleeding diathesis could be highly heterogeneous. According to the World Federation of Hemophilia (WFH), the most prevalent RBDs are FXI and FVII deficiencies, with frequencies of 37% and 23% of the total RBDs, respectively. Fibrinogen disorders and FV deficiencies are 10% each; Deficiencies of FX and FXIII are 9% and 6% respectively. Combined FV + FVIII (3%) and FII (2%) deficiencies are reported to be the most rare bleeding disorders [2].

The Indian population is extremely heterogeneous. Over centuries, the gene-pool in different population groups has well segregated and fixed. Moreover, consanguineous marriage is still a common practice in some of the population groups, accounting for about 6–40% of all marriages [3]. As a result of this, the autosomal recessive bleeding disorders, which are of rare occurrence across the world, may not be as rare in many Indian population groups. As there is no systematic account of molecular pathology of RBDs from this vast country of continental dimension with approximately 1270 million people, it is but rational to attempt such a systematic analysis.

The present study addresses the molecular pathology of different RBDs in various parts of this country extracted from published literature and compares it with the existing data on RBDs reported in the world literature. It is thus a narrative account of RBDs from India as compared to that of other parts of the world.

## Materials and Methods

### Protocol

The review protocol involves study of all existing published literature on molecular pathology of RBDs from India. As every relevant report in published English literature on RBDs from India has been included, the study is holistic and comprehensive.

### Eligibility criteria and Information source

Studies describing molecular pathologies in RBDs in human patients (case series, review, original papers, were used to compile the present report).

### Search

To search the published literature from India on the molecular pathology of RBDs, Pubmed search was used (www.pubmed.com). To compare the distribution of the types of mutations reported in Indian studies, with that of world literature, the Human Gene Mutation Database (HGMD) was referred up to August 2013 (www.hgmd.org). The key words used for pubmed search were: “rare bleeding disorders”, “mutations”, “ India”, “fibrinogen”, “afibrinogenemia”, “factor II deficiency”, “prothrombin”, “factor VII deficiency”, “factor V deficiency”, “factor X deficiency”, “factor XI deficiency”, “combined factor V and VIII deficiency”, “factor XIII deficiency”, “Bernard Soulier syndrome” and “Glanzmanns thrombasthenia” in different combinations. A total of 60 relevant articles were returned using these key words (Flow Diagram S1).

### Data Collection process

The data was tabulated in master sheets, which were subsequently tabulated separately for each RBD. To keep the presentation uniform throughout, the reporting format of the mutations/polymorphisms was converted in accordance to the HGVS nomenclature, wherever possible.

### Data Items

The following data items were collected: deficiency, inheritance, bleeding, complications, laboratory findings, molecular pathologies in congenital/inherited fibrinogen disorders, FII, FV, FV+FVIII, FVII, FX, FXI, FXIII deficiencies and platelet disorders, GT and BSS.

### Risk of bias in individual studies and across studies

As all the studies are hospital based, the main bias is that, symptomatically milder patients may have been missed unless a family study has been done where one patient had severe manifestations.

### Additional analysis

As this study is a descriptive additive data, it did not require any additional analysis.

## Results

The total number of patients with RBDs and the mutations and polymorphisms reported from these patients has been compiled in Tables 1, 2, 3, 4, 5, 6, 7, 8, 9, 10. The percentage distribution of various types of mutations in RBDs in Indian patients is compared with that of the world literature as reported in the Human Gene Mutation Database (Table 11). The mutations and polymorphisms collectively reported from the Indian literature on RBDs were analyzed, and the observations for each disorder are summarized as under:

**Table 1:** Mutations in Indian patients with fibrinogen deficiency.

Sr. no.	Patient nos.	Gene/Location	Nucleotide change	Amino acid change	Comments	References
1	1	*FGB*, Exon 7	c.1241delG	§p.Gly414fs*2	Homozygous	[4]
2	2,12,24,25,26,27	*FGA*	§$c.G364+1A	-	Homozygous	
3	3,4,5,6,8,13,19,22	*FGG*, Exon 6	$c.554delA	$p.Lys185fs*13	Homozygous	
4	7	*FGB*, Exon 6	c.G862A	§p.Gly288Ser	Homozygous	
5	9	*FGA*, Exon 4	c.C381A	§ p.Tyr127*	Homozygous	
6	10	*FGA,* Exon 5	c.1725delA	§p.Lys575fs*74	Homozygous	
7	11,23	*FGB*, Exon 8	c.G1334C	§p.Arg445Thr	Homozygous	
8	14	*FGA*, Exon 5	c.1398delT	§p.Thr466fs*17	Homozygous	
9	15	*FGG*, Exon 6	c.243delA	§p.Ser81fs*5	Homozygous	
10	16	*FGG*, Exon 7	c.834_835delCT	§p.Asp278–279fs*17	Homozygous	
11	17	*FGB*	§c.G851+1A	-	Homozygous	
12	18	*FGA,* Exon 5	c.786_789delGAGA	p.Glu262–263fs*158	Homozygous	
13	20	*FGA*, Exon 5	c.887_894dup7	§p.Asp296fs*59	Homozygous	
14	21	*FGA*	§c.T510+2G	-	Homozygous	
15	28	*FGB*, Exon 8	c.G8017A	§,p.Gly434Asp	Homozygous	[5]

§ Novel variations, $ Common variations.

**Table 2:** Mutations in Indian patients with Factor II deficiency.

Sr. no.	Patient Nos.	Exon/Intron	Nucleotide change	Amino acid change	Comments	References
1	1	Exon 9	c.7484/7489Del GAA	p.Lys345del	Homozygous	[6]
		Intron E	g.T4048C	-	Homozygous	
2	2	Exon 2	c.G514A	p.Arg44Gln	Heterozygous	
		Exon 10	c.G8825A	§p.Ala405Thr	Heterozygous	
						
		Intron A	g.459Del T		Homozygous	
		Exon 2	c.A554G	p.Leu56Leu	Homozygous	
		Intron E	g.T4048C	-	Homozygous	
		Intron F	g.G4272A	-	Homozygous	
		Intron F	g.A4282G	-	Homozygous	
		Intron F	g.A4291G	-	Homozygous	
		Intron F	g.A4298G	-	Homozygous	
		Intron F	g.4304InsG	-	Homozygous	
3	3	Exon 8	c.C7311T	p.Arg314Cys	Homozygous	
		Intron E	g.T4048C	-	Homozygous	
		Intron E	g.C4125G	-	Homozygous	
4		Exon 6	c.C4203T	p.Thr165Met	Homozygous	
		Intron F	g.G4272A	-	Homozygous	
		Intron F	g.A4291G	-	Homozygous	
		Intron F	g.A4298G	-	Homozygous	
		Intron F	g.4304Ins G	-	Homozygous	
5	4	Exon 9	c.G7509A	p.Glu352Lys	Homozygous	
		Intron E	g.T4048C	-	Homozygous	
6	1	Exon 4	c.G269C	§p.Cys90Ser	Homozygous	[7]

§ Novel variations.

**Table 3:** Mutations in Indian patients with Factor V deficiency.

Sr. no.	Patient Nos.	Exon/Intron	Nucleotide Change	Amino acid change	Comments	Reference
1	1,2	13	§g.50936–50937	p.Pro835fs*	Homozygous	[8]
			delAA or AG, c.2662–2663			
2	3,4	13	§g.51660delA, c.3386delA	p.Ser1142fs*	Homozygous	
3	5	13	§g.52162delC, c.3888delC	p.Ser1259fs*	Homozygous	

§ Novel variations.

**Table 4:** Mutations in Indian patients with combined Factor V and Factor VIII deficiencies.

Sr. no.	Patient Nos.	Gene/Location	Nucleotide change	Amino acid change	Polymorphisms	Reference
1	1	*MCFD2*, **Exon 4**	§c.A365T	p.Asp122Val	c.764–10delT	[9]
2	2	*MCFD2*, Intron 2	$c.G149+5A	-	c.A351G (p.Arg117Arg)	
				-	c.539+11delGT	
				-	c.764–10delT	
	3, 4	*MCFD2*, Intron 2	$c.G149+5A	-	c.764–10delT	
	5	*MCFD2*, Intron 2	$c.G149+5A	-	c.A351G (p.Arg117Arg)	
		*MCFD2*		-	c.539+11delGT	
		*MCFD2*		-	c.764–10delT	
	6	*MCFD2*, Intron 2	$c.G149+5A	-	c.764–10delT	
3	7,8	*MCFD2*, Exon 3	§$c.210_244del35	p.Glu71fs	c.764–10delT	
4	9	*MCFD2*, Intron 2	IVS2+5(Homo)	-	-	
	10	*MCFD2*, Intron 2	IVS2+5(Homo)	-	-	
5	1	*LMAN1*, Exon 7	§c.813_822+62del72	p.Lys302fs	c.764–10delT	[9]
6	2	*LMAN1*, Exon 2	c.G340T	p.Gly114*	-	[10]

§ Novel variations, $ Common variations.

**Table 5:** Mutations in Indian patients with Factor VII deficiency.

Sr. no.	Patient nos.	Exon	Nucleotide change	Mutation	Comments	Polymorphisms	References
1	1	8	c.G904A	p.Asp302Asn	Heterozygous	-	[12]
2		8	c.A1223G	p.His408Arg	Heterozygous	-	
3	1	8	c.C859T	§$p.Gln287*	Homozygous	-	[11]
4	2,9	1a		§$p.Leu55fs	-	-	
5	3	2	c.C175T	§ p.Arg59Cys	Homozygous	-	
6	4,8	7	c.C752A	§ p.Ala251Glu	Homozygous	-	
7	5,6,7	8	c.C1324T	p.Gln442*	-	—	
8	8	8	c.G1272C	p.Trp424Cys	Homozygous	-	
9	10	5	c.G529C	p.Gly177Arg	Homozygous	-	
10	11	6	c.G635A	p.Arg212Gln	Homozygous	-	
11	1	2	c.T244C	p.Cys82Arg	-	-	[13]
12		6	c.G751C	§p.Ala251Pro	-	-	
13	2,3	6	c.G635A	p.Arg212Gln	-	-	
14	4	8	c.T968G	§p.Leu323Arg	Heterozygous	c.G10976A p.Arg413His Homo	
15	5	6	c.G529C	p.Gly177Arg	-	c.G10976A p.Arg413His Homo	
			c.T968G	§p.Leu323Arg	Heterozygous	-	
16	6	6	c.C1194G	§p.Asp398Glu	-	-	
17	7	8	c.G1109T	p.Cys370Phe	-	-	
18	8	8	c.C1151T	p.Thr384Met	-	c.7880T p.His175His Homo	
	9	8	c.C1151T	p.Thr384Met	-	-	
19	10	6	c.T593C	§p.Ile198Thr	-	c.G10976A p.Arg413His Homo	
		8	c.T968G	§p.Leu323Arg	Heterozygous	-	
	11,12	8	c.G1109T	p.Cys370Phe	-	c.T122C,c.G73A promoter	
20	13	8	c.A1223G	p.His408Arg			
21	14	8	c.T1030C	§p.Trp344Arg	Heterozygous	c.C7880T p.His175His Homo	
			-	-	-	c.G10976A p.Arg413His Homo	

§ Novel variations, $ Common variations.

**Table 6:** Mutations in Indian patients of Factor X deficiency.

Sr. no.	Patient nos.	Exon	Nucleotide change	Mutation	Comments	Polymorphisms	References
1	1, 11	7	c.T863C	§p.Val288Ala	Homozygous	c.C793T, p.Thr264Thr	[15]
2	2,3	8	c.G1216A	p.Gly406Ser	Homozygous		
3	4,5,7	1	c.C44A	§p.Ala15Asp	Homozygous	§c.T897C, p.Gly299Gly	
		1	c.C44A	§p.Ala15Asp	Homozygous	§c.T897C, p.Gly299Gly	
4	6	8	c.T1354A	p.Ile451Phe	Homozygous		
		1	c.C44A	§p.Ala15Asp	Heterozygous	§c.T897C, p.Gly299Gly	
5	8	4	c.G295C	§p.Gly99Arg	Homozygous		
6	9	7	c.A854T	§p.Lys285Met	Homozygous	c.C793T, p.Thr264Thr	
7	10	1	c.G61A	p.Gly21Arg	Homozygous		
-	12					c.C793T, p.Thr264Thr	
						c.C-40T, c.C-221A	
						c.T-223A	
8	13	8	c.T1061G	§p.Met354Arg	Heterozygous	c.C793T, p.Thr264Thr	
						c.C-40T	
9	14	1	c.G22T	§p.Val8Phe	Heterozygous		
	15					c.C793T, p.Thr264Thr	
	16	1	c.G22T	§p.Val8Phe	Double	c.C793T, p.Thr264Thr	
10		8	c.C1069T	§p.Gln356*	Heterozygous		
11	1	6	c.G517A	§p. Gly173Arg	Homozygous		[16]
			IVS1(-52)insCCTCTT		Homozygous		
			CACCCAGGCT				
12	2	8	c.A1180C	§p.Ser394Arg	Homozygous		
					Homozygous	c.C793T, p.Thr264Thr	
			IVS1(-52)insCCTCTT		Homozygous		
			CACCCAGGCT				
			IVS7A+33G		Homozygous		
13	3	7	c.G787A	§p.Gly263Arg	Homozygous		
					Homozygous	c.C793T, p.Thr264Thr	
			IVS3C+98A		Homozygous		
			IVS7A+33G		Homozygous		
14	4	2	c.T212C	§p.Phe71Ser	Heterozygous		
15		6	§c.514delT	§p.Cys172fs	Heterozygous		
			§c.T516G		Heterozygous		
			IVS2T-17C		Homozygous		
			IVS3C+98A		Homozygous		
16	5	8	c.G1087A	p.Gly363Ser	Homozygous		
	6	8	c.G1216A	p.Gly406Ser	Homozygous		
17	7	8	c.C1073T	p.Thr358Met	Homozygous		
			IVS1(-52)insCCTCTT		Homozygous		
			CACCCAGGCT			,	
			IVS3C+98A		Homozygous		

§ Novel variations.

**Table 7:** Mutations in Indian patients with Factor XI deficiency.

Sr. no.	Patient nos.	Exon	Nucleotide change	Mutations	Comments	Polymorphisms	Reference
1	1	8	c.G865C	§Val271Leu	Heterozygous	Int A, g.A-138C int A Het	[17]
2		12	c.G1433C	Gly460Arg	Heterozygous	p.Gly379Gly Het	
	2*	12		Gly460Arg	Homozygous	Int A, g.A-138C int A Homo	
						p.Gly379Gly Homo	
3	3	10	c.A1106C	§Tyr351Ser	Homozygous		

§ Novel variations.

* Patient 3 also showed a mutation p.Phe349Val in *F9* gene.

**Table 8:** Mutations from Indian patients with Factor XIII deficiency.

Sr no.	Patient nos.	Exon	Nucleotide change	Amino acid change	Comments	Polymorphisms	References
1	1	4	c.C523T	p.Arg174*	Homozygous	IVS1, g.A246G	[20]
2	2	3	§c.C267T	§p.Gln85*	Homozygous	IVS1, g.A246G, §A6T	
3	3	5	§c.A689G	§p.Gln229Arg	Homozygous	IVS1, g.A246G, Exon8, p.Pro331Pro	
4	4	14	c.G2045A	p.Arg681Gln	Homozygous	IVS1,g.A246G, p.Val650Leu Homo, Ex14	
5	5	6	c.G782A	p.Arg260His	Homozygous	Exon12 p.Pro564Leu	
6	6	2	§c.T58C	§p.Ser19Pro		Exon12 p.Pro564Leu	
7	7	4	§c.521delG		Heterozygous	IVS1,g.A246G	
8		6	§c.T790C	§p.Ser263Pro	Heterozygous	-	
9	1,6	10	c.G1243T	p.Val414Phe	Homozygous	Intron7 g.973+26delT; g.973+50insC	[21]
10	2	3	§c.T210G	§p.Tyr69*	Homozygous	c.G103T, p.Val34Leu; c.C1794T, p.Pro564Leu;	
11	3	6	§c.C791T	§p.Ser263Phe	Heterozygous	c.G1951A, p.Val650Ile; c.G1954C, p.Glu651Gln	
12		Intron14	§g.G2045A-1		Heterozygous	G-246A, c.T1216_23C, c.C1216_24T	
13	4	10	c.C1241T	p.Ser413Leu	Homozygous		
	5	6	c.G789T	p.Arg260His	Homozygous		
	6	10	c.G1243T	p.Val414Phe	Homozygous		
14	7	7	§c. 892_895dupG	§p.ser290_Ala291fs	Homozygous		
15	8	12	§c.1642_1644dupA	§p.Tyr547fs	Homozygous		

§ Novel variations.

All the mutations reported above are reported in *F13A* gene.

**Table 9:** Mutations and polymorphisms in Indian patients of Glanzmann's Thrombasthenia (GT).

Sr. no.	Patient nos.	Gene/Location	Nucleotide change	Amino acid change	Comments	Polymorphisms	References
1	1,2,3,4,5	*ITGA2B*, Promoter	§g.G951A	-	Homozygous	-	[27], [28]
2	1,2,4,5,6	*ITGA2B*, Exon 12	§c.T1028C	p.Leu343Pro	Homozygous	-	
3	7,8,9	*ITGA2B*, Exon 6	c.T641C	p.Leu214Pro	Homozygous	-	
4	10	*ITGA2B*, Exon 10	§c.G937A	p.Ala313Thr	Homozygous		
5	11	*ITGA2B*, Exon 12	c.G1073A	p.Arg358His	Homozygous	-	
6	12	*ITGA2B*, Exon 13	c.G1234A	p.Gly412Arg	Homozygous	-	
7	13	*ITGB3*, Exon 2	§c.G92A	p.Cys31Tyr	Homozygous	-	
8	14	*ITGB3*, Exon 3	c.G356A	p.Arg119Gln	Homozygous	-	
9	15	*ITGB3*, Exon 5	c.G752A	p.Arg242Gln	Homozygous	-	
10	16	*ITGB3*, Exon 7	§c.T953C	§p.Leu318Ser	Homozygous	-	
11	17	*ITGB3*, Exon 7	§c.A1031G	§p.Tyr344Cys	Homozygous	-	
12	18	*ITGB3*, Exon 10	§c.C1641G	§p.Cys547Trp	Homozygous	-	
13	19	*ITGB3*, Exon 15	§c.T2315C	§p.Leu772Pro	Homozygous	-	
14	20	*ITGB3*, Exon 4	§c.G415C	§p.Asp139His	Homozygous	-	
15	20	*ITGB3*, Exon 4	c.A422G	p.Tyr141Cys	Homozygous	-	
16	21	*ITGB3*, Exon 1	§c.T59C	§p.Leu20Pro	Homozygous	-	
17	22	*ITGA2B*, Exon 4	§c.559delG	§p.187, fs223*	Homozygous	-	
18	23,24	*ITGA2B*, Exon 19	§c.1919_1920delTG	§p.640, fs659*	Homozygous	-	
19	25	*ITGA2B*, Exon 23	§c.233delG	p.780, fs909*	-	-	
20	26	*ITGA2B*, Exon 26	§c.2674_2675insGA	p.892, fs910*	-	-	
21	2	*ITGA2B*,Exon 28	c.2915_2916insC	p.972, fs1035*	Homozygous	-	
22	27	*ITGA2B*, Exon 30	§c.3117_3118insTGGAG		Homozygous	-	
23	28,26	*ITGA2B*, Exon 14	§c.1424_1427dupAGGT	p.476, fs661*		-	
24	29	*ITGB3*, Exon 5	§c.674delA	p.225, fs282*		-	
25	30	*ITGB3*, Exon 14	§c.2217delC	p.740,fs		-	
26	31,32	*ITGB3*, Exon 6	§c.887_901delACGGGCA	p.296-300del		-	
			GTGTCATG			-	
27	33	*ITGB3*, Exon2	§c.155_156delGCinsTT			-	
28	34	*ITGA2B*	g.-2A800T	Splice site	Het/Missense	-	
29	34	§ *ITGA2B*	c.A1210+4G	Splice site	Het/Missense		
30	35	*ITGA2B*	c.G1753-1A	Splice site	Het/Missense	-	
31	36	*#ITGA2B*	c.188+8delG	Splice site	Het/Deletion	-	
32	1	§ *ITGB3*, Exon 3	c.T465C	p.Ser149Pro	Homozygous	-	[24], [25]
33	2	§ *ITGB3*, Exon 3	c.A13839G	p.Tyr141Cys	Homozygous	-	
34	3	§ *ITGB3*, Exon 5	c.16666(+T)	fs	Homozygous	-	
35	4	§ *ITGB3*, Exon6	c.A19095C	p.Met321Leu	Homozygous	-	
36	5,7,8,9	§ *ITGB3*, Exon 4	c.15739(-C)	fs	Homozygous	-	
37	6	§ *ITGB3*	c.C14065T	Stop codon	Homozygous		
38	10	§ *ITGB3*, Exon 4	c.15841/42(+GG)	Dupl/Ins fs	Homozygous	-	
39	11	§ *ITGB3*, Exon 4	c.15846 (+G)	fs	Homozygous	-	
40	12	§ *ITGB3*, Exon 4	c.15790(-C)	fs	Homozygous	-	
41	13	§ *ITGB3*, Exon 4	c.15762(+T)	fs	Homozygous	-	
42	1	§ *ITGB3*, Exon 10	c.1595G_A	p.Cys532Tyr	Homozygous	-	[26]
43	2	§ *ITGB3*, Exon 2	c.126-129 Ins AGTG		Homozygous	-	
44	1	*#ITGA2B*, Exon 12	c.G1186A	p.Asp396Asn	Homozygous	c.C3063T exon 30, g.CIVS21(-7)G, c.T2621G exon 26	[27]
45	2	*#ITGA2B*, Exon 17	c.G1652A	p.Arg551Trp	Homozygous	-	
46	3	*#ITGA2B*, Exon 2	g.GIVS2(+1)A	Alt splicing	Homozygous	c.C3063T exon 30, g.CIVS21(-7)G, c.T2621G exon 26	
47	4	*#ITGA2B*, Exon 22	c.G2264C	p.Arg755Pro	Homozygous	c.C3063T exon 30, g.CIVS21(-7)G, c.T2621G exon 26	
48	5	*#ITGA2B*, Exon 9	c.G859C	P.Gly287Arg	Homozygous	-	
49	6	*#ITGA2B*, Exon 13	c.C1374G	p.Ile458Met	Homozygous	-	
50	7	*#ITGA2B*, Exon 23	c.C2315G	p.Pro772Arg	Homozygous	-	
51	8	*ITGA2B*, Exon 12	1073delGTGT	fs	Homozygous	-	
52	9	*ITGA2B*, Exon 13	1230delC	fs	Homozygous	-	
53	10	*#ITGA2B*, Exon 24	2415delCA	fs	Homozygous	-	
54	11,12	*#ITGA2B*, Exon 16	g.GIVS16(+1)A	Alt splicing	Homozygous	c.C3063T exon 30, g.CIVS21(-7)G, c.T2621G exon 26	
55	13	*#ITGA2B*, Exon 26	267delA	fs	Homozygous	c.C3063T exon 30, g.CIVS21(-7)G, c.T2621G exon 26	
56	1	*ITGA2B*, Exon 1	c.G48A	Trp47*	Homozygous	*ITGA2B*,IVS16(+11)T>C	[22], [23]
57	2	*ITGA2B*	c.G475A	§p.Gly159Ser	Homozygous	*ITGB3*,5'UTR(-35)InsG; *ITGB3*,5'UTR(-35)InsG	
58	3	*ITGA2B*	c.C953T	§p.Ser318Leu	Homozygous		
59	4	*ITGA2B*	c.G1162A	§p.Gly388Ser	Homozygous	*ITGB3*,5'UTRG(-114)C; *ITGB3*,5'UTR(-35)InsG; *ITGB3*, UTR(-24)G>C	
60	5	*#ITGA2B*	g.GIVS15(-1)A	Alt splicing	Homozygous	*ITGA2B*,g.CIVS21(-7)G *ITGA2B* c.T2622G (p.Ile874Ser)	
						*ITGA2B* c.C3063T(p.Val1021Val)	
						*ITGB3*,5'UTR(-35)InsG	
61	6,7	*ITGA2B*	c.C1651T	§p.Arg582Trp	Homozygous	*ITGB3*,5'UTRG(-114)C; *ITGB3*,5'UTR(-35)InsG	
						*ITGB3*,5'UTR(-24)G>C	
						*ITGB3*, c.T176C (p.Leu59Pro)(HT); *ITGB3*, c.G1533A (p.Glu511Glu)(HT)	
						*ITGB3*, c.A1545G(p.Arg515Arg)(HT); IVS10(+23)C>G(HT)	
62	8	*ITGA2B*, Exon 22	c.G2236T	§p.Glu746*	Homozygous	*ITGA2B*,g.CIVS21(-7)G; *ITGA2B* c.T2622G (p.Ile874Ser)	
						*ITGA2B*, c.C3063T (p.Val1021Val)	
63	9	*ITGA2B*	c.T2489G	§p.Leu830Arg	Homozygous	*ITGA2B*,g.CIVS21(-7)G; *ITGA2B* c.T2622G (p.Ile874Ser)	
						*ITGA2B*,g.IVS26G(+22)T; *ITGA2B*, c.C3063T (p.Val1021Val)	
						*ITGB3*,5'UTRG(-114)C(HT); *ITGB3*5'UTR(-35)InsG	
						*ITGB3* c.C1641T(HT)	
64	10	§ *ITGB3*	c.330-336TCCCCAGdel		Homozygous	*ITGA2B*,g.CIVS21(-7)G; *ITGA2B* c.T2622G (p.Ile874Ser)	
						*ITGA2B*,g.IVS26G(+22)T; *ITGA2B*, c.C3063T (p.Val1021Val)	
						*ITGB3*,c.T176C (p.Leu59Pro)	
65	11	*ITGB3*, exon 11	c.T1801G	§p.Cys601Gly	Homozygous	*ITGA2B*,g.CIVS21(-7)G	
						*ITGA2B*,c.T2622G (p.Ile874Ser)	
						*ITGA2B*,g.IVS26G(+22)T	
						*ITGA2B*,c.C3063T (p.Val1021Val)	

fs. Frameshift mutations; § Novel variations.

**Table 10:** Mutations in Indian patients with Bernard Soulier Syndrome (BSS).

Sr. no.	Patient nos.	Gene/Location	Nucleotide change	Amino acid change	References
1	1,5,6,13,14,18	*GP9*, Exon 3	c.T70C	$p.Cys24Arg	[30]
2	2,8,9,12,16,17,19,21,23,24	*GP1BB*, Exon 2	c.124_145del22bp	$§p.Arg42del	
3	3	*GP9*, Exon 3	c.119delG	§p.Gly40del	
4	4	*GP1BB*, Exon 2	c.G462C	§p.Gln154His	
5		*GP1BB*, Exon 2	c.T470C	§p.Leu157Pro	
		*GP1BA*, Exon 2	c.T1522C	§p.Tyr508His(het)	
6	7	*GP9*, Exon 3	c.437_474dup38	§p.Pro146*	
7	10	*GP1BB*, Exon 2	c.C269G	§p.Pro90Arg	
8	11,26	*GP9*, Exon 3	c.T212C	§p.Phe71Ser	
9	15,20	*GP9*, Exon 3	c.T212G	§p.Phe71Cys	
10	22	*GP1BA*, Exon 2	c.1253_1257dupC	p.Pro418del	
11	25	*GP1BA*, Exon 2	c.339InsGA	§p.Gln113del	
12	27	*GP9*, Exon 3	c.C328T	§p.Gln110*	
13	1	*GP9*, Exon 3	c.T285G	§p.Tyr95*	[31]
14	2,3	*GP9*, Exon 3	c.T70C	$p.Cys24Arg	
15	4	*GP1BA*, Exon 2	c.1013InsA	§p.Met338del	
16	5,6	*GP1BA*, Exon 2	c.C97A	p.Cys32*	
17	7	*GP1BA*, Exon 2	c.1455InsT	p.Val485del	
18	8	*GP1BA*, Exon 2	c.G404A	§p.Cys135Tyr	
19	1	*GP1BA*, Exon 2	c.G138A	p.Trp46*	[32]
20	2	*GP1BA*, Exon 2	c.236dupA	§p.Asp79del	
21	3	*GP1BA*, Exon 2	c.941dupT	§p.Phe314del	
22	4	*GP1BA*, Exon 2	c.278dupC	§p.Pro93del	
23	5	*GP1BA*, Exon 2	c.266dupA	§p.Asp89del	
24	6	*GP1BA*, Exon 2	c.1465delG	§p.Glu489del	
25	7	*GP9*, Exon 3	c.T285G	§p.Tyr95*	
26	8	*GP1BA*, Exon 2	c.1064dupT	§p.Phe355del	
27	9,10	*GP9*, Exon 3	c.T70C	$p.Cys24Arg	
28	11	*GP1BA*, Exon 2	c.143dupT	§p.Leu479del	
29	12,13	*GP1BA*, Exon 2	c.T785G	p.Val262Gly	
30	14,15	*GP1BA*, Exon 2	c.1592delT	§p.Leu515del	

§ Novel variations, $ Common variation.

**Table 11:** Comparison of percentage distribution of Indian RBD mutations with HGMD mutations.

	*FGA/FGB/FGG*		*F2*		*F5*		*LMAN1*		*MCFD2*			
	HGMD	Indian	HGMD	Indian	HGMD	Indian	HGMD	Indian	HGMD	Indian		
**Missense/Nonsense**	152(67.25%)	4(26.66%)	42(76.36%)	5(83.33%)	61(58.65%)		10(29.41%)	1(50%)	9(50%)	3(75%)		
**Splicing**	17(7.52%)	3(20%)	3(5.45%)		11(10.57%)		6(17.64%)		3(16.66%)			
**Regulatory**	7(3.09%)		4(7.27%)		0		0		0			
**Small deletions**	29(12.83%)	8(53.33%)	4(7.27%)		23(22.11%)	3(100%)	13(38.23%)	1(50%)	3(16.66%)	1(25%)		
**Small insertions**	9(3.98%)		1(1.81%)	1(16.66%)	7(6.73%)		4(11.76%)		1(5.55%)			
**Small indels**	4(1.76%)		0		0		0		0			
**Gross deletions**	7(3.09%)		1(1.81%)		1(0.96%)		1(2.94%)		2(11.11%)			
**Gross insertions/duplications**	1(0.44%)		0		0		0		0			
**Complex rearrangements**	0		0		1(0.96%)		0		0			
**Repeat variations**	0		0		0		0		0			
**Total mutations**	**226**	**15 (6.63%)**	**55**	**6 (10.90%)**	**104**	**3 (2.88%)**	**34**	**2 (5.88%)**	**18**	**4 (22.22%)**		

	*F7*		*F10*		*F11*		*F13*		*GP1BA/GP1BB/GP9*		*ITGA2B/ITGB3*	
	HGMD	Indian	HGMD	Indian	HGMD	Indian	HGMD	Indian	HGMD	Indian	HGMD	Indian
**Missense/Nonsense**	162(63.03%)	20(95.23%)	85(82.52%)	16(94.11%)	179 (80%)	3 (100%)	57(58.16%)	11(73.33%)	24(57.14%)	16(53.33%)	136(57.14%)	35(53.84%)
**Splicing**	38(14.78%)		5(4.85%)		19 (8%)		10(10.20%)	1(6.66%)	0		31(13.02%)	7(10.76%)
**Regulatory**	19(7.39%)		0		2 (1%)		0		2(4.76%)		1(0.42%)	
**Small deletions**	23(8.94%)	1(4.76%)	8(7.76%)	1(5.88%)	13 (6%)		17(17.34%)	1(6.66%)	12(28.57%)	11(36.66%)	39(16.38%)	13(20%)
**Small insertions**	5(1.94%)		0		7 (3%)		8(8.16%)		2(4.76%)	3(10.00%)	20(8.40%)	8(12.30%)
**Small indels**	2(0.77%)		1(0.97%)		2 (1%)		1(1.02%)		0		4(1.68%)	1(1.53%)
**Gross deletions**	2(0.77%)		4(3.88%)		3 (1%)		4(4.08%)		2(4.76%)		2(0.84%)	
**Gross insertions/duplications**	0		0		0		0	2(13.33%)	0		2(0.84%)	1(1.53%)
**Complex rearrangements**	5(1.94%)		0		0		1(1.02%)		0		3(1.26%)	
**Repeat variations**	1(0.38%)		0		0		0		0		0	
**Total mutations**	**257**	**21 (8.17%)**	**103**	**17 (16.50%)**	**225**	**3 (1.33%)**	**98**	**15 (15.30%)**	**42**	**30 (71.42%)**	**238**	**65(27.31%)**

Percentage of mutations detected in different genes in Indian patients (184 mutations) versus HGMD is as follows: FGA/FGB/FGG 8.15% (HGMD 16.14%; *F2* 3.26% (HGMD 3.92%); *F5* 1.63% (HGMD 56.5%); *LMAN1* 1.08% (HGMD 2.42%; *MCFD2* 2.17% (HGMD 1.28%) *F7* 8.15% (HGMD 16.14%); *F10* 10.32% (HGMD 7.35%); *F11* 1.63% (16.07%); *F13* 8.69% (HGMD 7%); *GP1BB/GP9*: 16.3% (HGMD 3%); *ITGA2B/ITGB*: 35.32% (HGMD 17%).

### Fibrinogen deficiency

Mutations in three genes (*FGA, FGB, FGG*) encoding fibrinogen α-, β- and γ-chains lead to congenital fibrinogen deficiency, which is an extremely rare hereditary bleeding disorder, affecting 1 in 1,000,000 individuals [4], [5]. There are two reports of molecular characterization of fibrinogen deficiency in Indian patients which collectively report the molecular pathology of 28 patients [4], [5]. The first report of “Fibrinogen Mumbai” describes a novel homozygous c.G8017A transition in exon 8 found in *FGB* gene. The resulting p.G434D missense mutation involves a highly conserved amino acid residue, located in the C-terminal globular D domain. This p.G434D substitution causes severe hypofibrinogenemia by impairing fibrinogen secretion as confirmed by expression data [5]. Another study describes a series of 27 patients with fibrinogen deficiency and the underlying molecular pathologies [4].

Fibrinogen alpha (*FGA*), beta (*FGB*), gamma (*FGG*) genes collectively were found to have a total of 15 disease causing mutations with 8 frameshift, 3 splice site, 3 missense and 1 nonsense mutation in 27 patients. 13 of them were novel; 7 were frame shift (*FGA*: p.Asp296fs*59, p.Thr466fs*17 and p.Lys575fs*74; *FGB*: p.Gly414fs*2 and *FGG*: p.Ser81fs*5, p.Lys185fs*13 and p.Asp278_279 fs*17), 3 splice site mutations (*FGA* c. G364+1A; c.T510+2 G; *FGB* c.G851+1A), 2 missense substitutions (*FGB* p.Gly288Ser; p.Arg445Thr); a nonsense mutation in *FGA* (p.Tyr127*) and two common mutations (*FGA*: c. G364+1A, *FGG*: p.Lys185fs*13) in 14 patients have been reported [4]. *FGG*: p.Lys185fs*13 is the most common mutation observed in one third of the Indian patients, while the second mutation, a splice site variant i.e. *FGA*: c.G364+1A was observed in 5 out of 27 patients (18.5%) analyzed. This finding is significant as it provides a first line screening strategy for mutation detection in this gene in Indian patients. The nucleotide and the resultant amino acid variations reported are summarized in Table 1.

### Prothrombin deficiency

Prothrombin (coagulation factor II) is the precursor of thrombin, a serine protease in the coagulation cascade. The Prothrombin gene is located on chromosome 11 (11p11–q12 [2]. Prothrombin deficiency occurs in approximately 1: 2,000,000 individuals, with diverse molecular basis. The mutations or polymorphisms in *F7* gene are known to cause both thrombosis and bleeding [6], [7]. Six mutations have been reported by different groups from the Indian literature so far, which lead to prothrombin deficiency [6], [7]. Two of these mutations are novel; p.Ala405Thr change affecting ‘B’ chain of α-thrombin i.e. Prothrombin Vellore located in the Histidine disulfide loop of the thrombin B chain [6] and the other one, Prothrombin Mumbai i.e. c.G269C mutation which caused a p.Cys90Ser substitution in the primary protein [7]. Mutations in *F2* gene generally do not lead to complete absence of the protein as severe prothrombin deficiency is incompatible with life. The *F2* mutations reported from India are shown in Table 2.

### Factor V deficiency

Congenital factor V (FV) deficiency is generally associated with moderate to severe bleeding symptoms. In the world literature, a total of 104 mutations, located in the *F5* gene, have been described in patients with severe FV deficiency (http://www.hgmd.org/). Most of the mutations reported are localized to exon 13 of the *F5* gene. There is one report from the Indian literature so far, where mutations in 5 unrelated patients were reported, of which 3 were novel small deletions, present in homozygous state, g.50936–50937delAA or AG and g.51660delA, occurring in two different patients, and g.52162delC in another patient [8]. Interestingly, the mutations reported by this group were all found in exon 13. Screening for mutations in exon 13 should be the first step before proceeding to the remaining exons in this gene. Table 3 summarizes the mutations reported in this study.

### Combined FV and FVIII deficiency

Combined FV and FVIII deficiency is a rare autosomal recessive bleeding disorder occurring in 1: 1,000,000 individuals. Mutations in one of the two genes encoding the proteins i.e. lectin mannose binding protein (*LMAN1*) and multiple coagulation factor deficiency 2 (*MCFD2*) lead to deficiencies of FV and FVIII. 13 patients with combined FV and FVIII deficiency have been reported in the Indian literature by two different groups, where a complete molecular characterization has been done [9], [10]. In one of the families mutation was not detected in both the genes which suggests the existence of a third locus involved in the secretion pathway of FV and FVIII and associated with the combined deficiency [10]. The mutations reported by these groups are summarized in Table 4. Three of them, a 72 bp deletion in *LMAN1* (c.813_822+62del72, p.K272fs), a 35-bp deletion in *MCFD2* (c.210_244del35) and a missense mutation in *MCFD2* (p.D122V) were identified in 4 patients. A nonsense mutation, i.e. G to T substitution, in exon 2 of the *LMAN1* gene, was novel [9].

### Factor VII deficiency

FVII is a vitamin K-dependant serine protease synthesized in the liver and has a very important position in the coagulation cascade. Factor VII deficiency is a rare (1: 500,000) autosomal recessive disorder of blood coagulation caused by heterogeneous mutations (∼140) in *F7* gene [10]. The clinical features are quite variable in patients with FVII deficiency. The severity of bleeding is not well correlated with the FVII activity. Mutations in 26 patients with FVII deficiency have been characterized and 21 mutations and 6 polymorphisms have been reported from various studies from India [11]–[13]. Two mutations occurred in double heterozygous form in a patient, p. Asp302Asn and p. His408Arg substitution in the 8th exon of the *F7* gene. p.Asp302 is one of three absolutely conserved active-site residues found in all serine proteases, whereas p. His408Arg substitution is located in exon 8 of *F7* corresponding to the catalytic domain of the enzyme.

A total of 18 missense, 2 nonsense, and 1 frame shift mutation has been reported, of which 10 were novel variations. It is interesting to note that 15 of these 26 patients were found to have the disease causing mutations in exon 8; 7 had the mutations in exon 6 of the *F7* gene and 6 of the mutations were located in the region corresponding to the catalytic serine protease domain. Using haplotype analysis, it was shown that p.Leu55fs in the pro-peptide region and p.Gln287* in the catalytic domain which resulted in premature termination codon in two patients had a common founder. Successful prenatal diagnosis in the second trimester of pregnancy by cordocentesis, followed by FVII and other coagulation factor assays could be given to a family with one affected child [13].

Another study showed that functional polymorphisms in *F7* gene affect the phenotype of patients with severe Hemophilia. *F7*353Q allele was shown to be associated with a severe phenotype. *F7* Arg/Gln and Gln/Gln genotypes were found to be significantly higher in patients with severe phenotype when compared to patients with milder phenotype (P = 0.045) [14]. The mutations reported from Indian studies are presented in Table 5.

### Factor X deficiency

Factor X (FX) deficiency is a rare autosomal recessive bleeding disorder that is estimated to occur in 1: 100,000 individuals worldwide. The gene encoding *F10* is located on the long arm of chromosome 13, consists of 27 Kb of nucleotide sequence with eight exons and seven introns [15]. 103 variants, comprising deletions, missense, frameshift and splice site mutations have been reported in *F10* gene (http://www.hgmd.org/). Most mutations are missense and the common sites of mutations have been localized to exon 2 (Gla domain) and exons 7 and 8 (catalytic domains). Mutations in a total of 22 patients with FX deficiency from India and 1 from Nepal have been characterized and reported by Indian groups. 17 mutations and 5 polymorphisms have been reported, of which 15 are missense, 1 frame-shift and 1 nonsense mutation. 13 novel mutations and 1 novel polymorphism have been documented [15], [16]. Nine of the patients were found to have the mutation in exon 8. p.Gly406Ser which resulted in a cross reactive material positive phenotype with FX antigen levels similar to wild-type but undetectable activity was the disease causing mutation in 3 of the patients. The polymorphism c.C793T (p.Thr264Thr) was found in 9 patients from two independent study groups. Second trimester prenatal diagnosis by phenotypic factor assays in cord blood sample was given to a family with one child affected with FX deficiency [15]. The various mutations and polymorphisms studied in FX deficiency patients from India are summarized in table 6.

### Factor XI deficiency

Factor XI is present in the plasma as a zymogen. It exists as a homodimer consisting of two identical polypeptide chains linked by disulfide bonds. During activation, an internal peptide bond is cleaved by factor XIIa, resulting in activated factor XIa, which activates factor IX. Deficiency of FXI is referred to as Rosenthal syndrome or Hemophilia C. The prevalence of FXI deficiency in the world literature is estimated to be about 1 in 100,000 to 1 in 1 million. Though a few cases of FXI deficiency have been identified in the Indian population [18], [19], there is only one report on molecular characterization of FXI deficiency in 2 patients and one patient with combined FIX and FXI deficiency [17]. There are only 3 mutations reported in *F11* gene from India. One patient had a double coagulation defect, a novel c.T31166G transversion (p.Phe349Val) in *F9* corresponding to the catalytic domain of FIX, along with a homozygous p.Gly460Arg mutation in *F11* corresponding to the catalytic domain of FXI. Another patient was shown to have the p.Gly460Arg mutation in heterozygous state with a novel p.Val271Leu mutation that affected the apple-3 domain of FXI (Table 7) [17]. This mutation was predicted to abolish the physiological donor splice site and result in an abnormal FXI transcript. The third patient was detected to have a novel p.Tyr351Ser mutation in exon 10 of *F11* corresponding to the apple-4 domain in FXI. This group also reported 2 polymorphisms -138C in intron A and p.Gly379Gly in heterozygous state (Pt.1) or a homozygous state (Pt.2) in two patients (Table 7) [17].

### FXIII deficiency

Factor XIII is a plasma transglutaminase protein that is essential for normal haemostasis and fibrinolysis. FXIII deficiency is a rare autosomal (1: 2,000,000) recessive disorder of blood coagulation. Mutations are mostly reported in the *F13A* gene. FXIII deficiency leads to serious bleeding diathesis, the common symptoms being bleeding from the umbilical stump, prolonged bleeding post-injury, menorrhagia, poor healing of wounds, intra cranial bleed and spontaneous abortions. From the Indian studies, 15 patients of FXIII deficiency have been characterized for their molecular pathology. A total of 15 mutations have been identified, out of which 8 are missense, 2 duplications, 1 heterozygous deletion, 1 splice site and 3 nonsense mutations. Some polymorphisms were also identified, of which 1 was novel. Three mutations were detected in exon 10 in 4 patients and four mutations in exon 6 of *F13A*. The IVS1 A246G polymorphism was found to be present in 6 unrelated patients [20], [21]. It would be interesting to study the heterozygosity frequency of this polymorphism, which may be a useful marker for offering possible prenatal diagnoses for the affected families. The mutations and polymorphisms identified are presented in Table 8.

### Inherited disorders of platelet function

#### Glanzmanns Thrombasthenia (GT)

Glanzmanns thrombasthenia (GT) is a rare (1: 200,000) congenital autosomal recessive bleeding disorder caused by either lack or dysfunction of the platelet integrin αIIbβ3, encoded by genes *ITGA2B* and *ITGB3*. The integrin serves as a receptor for fibrinogen and von Willebrand factor along with some other plasma glycoproteins. The mutations that cause GT are spread all over the *ITGA2B* and *ITGB3* genes and more than 200 mutations have been reported from studies worldwide, which mainly comprise of deletions, point mutations, inversions, insertions and splice site variations [22]. The data published from Indian studies shows mutation analysis in 102 patients of GT, wherein mutations could be detected in 75 patients, out of which 47 were novel. A total of 65 mutations were detected which included 32 missense, 8 insertions, 3 nonsense, 13 deletions, 1 indel, 1duplication and 7 splice site variations. 17 polymorphisms were reported, of which 5 were novel. One of the studies showed that exon 4 of *ITGB3* is a common site for mutations to occur, where they have reported 5 deletions and 3 insertions [23]–[27]. Three polymorphisms were found to be in complete linkage disequilibrium i.e. g.CIVS21(-7)G, c.T2621G in exon 26, and c.C3063T in exon 30 of *ITGA2B*. The variations reported in Indian GT patients are documented in Table 9.

#### Bernard Soulier Syndrome (BSS)

Bernard–Soulier syndrome (BSS) is an extremely rare (1: 1,000,000) bleeding disorder of platelet adhesion, caused by defects in the glycoprotein genes *GP1BA* (encoding GPIbα), *GP1BB* (encoding GPIbβ) and *GP9* (GPIX) with autosomal recessive inheritance pattern. Molecular defects in any of the genes affect the expression of the glycoprotein complex on the platelet surface membrane. This leads to defective platelet adhesion and the patients with this defect manifests with muco-cutaneous bleeds, with giant platelets and thrombocytopenia. Only 42 mutations causing BSS have been reported in the world literature (http://www.hgmd.org/). From India, there are three reports by two groups on the molecular characterization of 50 BSS cases in which 30 disease causing mutations were identified. These included 6 nonsense, 10 missense, 3 insertion and 11 frameshift mutations, of which 25 were novel [29]–[31]. It is interesting to note that the p.Cys8Arg was found to be a common disease causing mutation in *GP9* by both the groups which had carried out independent studies on BSS patients [30]–[32] (Table 10). A second common mutation i.e.p.Arg42del has been found in 10 patients in one of the studies. All these patients were from the Southern states of India and may have a common founder. This data will make the job of molecular diagnosis of BSS patients from the Southern states of India much easier by screening for the common mutations first.

## Discussion and Conclusion

Several reports are available on the clinical and molecular pathologies of common bleeding disorders from across the globe over a period of time. However, information on the molecular pathologies of the rarer bleeding disorders is very limited because of their much lower frequency of occurrence. With the increasing number of patients presenting with RBDs, there is a need to investigate these disorders extensively for their clinical and molecular pathologies. The various molecular variations and defects and their resultant impact on coagulation proteins would give us valuable insights on the structure- function correlation in these proteins.

The types of mutations reported from Indian studies and their percentage distribution with respect to the total number of mutations are largely similar to the mutation data reported in the HGMD (Table 11). However, it is interesting to note that a much higher percentage of small deletions (53.33%) were found in fibrinogen deficiency patients as against 12.83% in world literature. Similarly, 22.11% of FV deficiency patients had small deletions, whereas all the 3 mutations found in Indian patients were small deletions. Two patients (13.33%) of FXIII deficiency had duplication mutations. In GT, 20% of the mutations were deletions, 12.3% insertions and 1.53% indels and duplications each, which are not reported in the literature. All the 3 mutations reported in factor XI deficiency patients from India were missense variations. One of the arguments however could be that the number of patients for each RBD from India is not big enough to get the true picture of their mutation distribution.

The genetic constitution of Indian population is different from that of the western populations. The population groups in India are largely closed groups where marriages happen within the same communities. Moreover, because of the practice of consanguineous marriages in some groups, one would expect much larger occurrence of autosomal recessive disorders, like the RBDs and inherited platelet disorders. Of the total mutations reported in the World literature, 71% in BSS, 27% in GT, 22% in combined FV an FVIII deficiency, 16.50% in FX deficiency, 1.33% in FXI deficiency, 15% in FXIII deficiency, 10% in FII deficiency, 8% in FVII deficiency, about 7% and 3% in fibrinogen disorders and FV deficiency respectively are reported from Indian patients (Table 11).

It is interesting to note that several novel mutations and polymorphisms are reported which have not only added valuable knowledge to the database but these can also be utilized to better understand the molecular pathologies and in designing improved diagnostic strategies for the RBDs. Some common mutations could be found in RBDs like fibrinogen deficiency, where 2 common mutations (*FGA*: c.G364+1A, *FGG*: p.Lys185fs*13) were found in 14 patients; 2 common mutations p.Leu55fs and p.Gln287* in two patients with FVII deficiency had a common founder. Two common mutations in BSS, i.e. p.Cys24Arg and p.Arg42del were found to have affected 8 and 10 of patients respectively. Two common mutations (p.Glu71fs, c.G149+5A) were identified in *MCFD2* gene in seven of nine patients [9], suggesting that this gene could be studied on preference for F5F8D. 8 mutations were localized to exon 4 of GPIIIa (*ITGB3*) in GT patients, 2 missense mutations in GPIIb (*ITGA2B*), g.G951A in the promoter and Leu343Pro in exon 12 were detected in 5 patients each. In patients with FVII deficiency, mutations were found largely in exon 6 and exon 8 and in the catalytic serine protease domain. A cost effective strategy can thus be adopted by screening these exons followed by the remaining exons. Similarly, in patients with Factor X and FXIII deficiencies, common sites of mutations were identified by independent study groups [15], [16], [20], [21]. Moreover, several novel polymorphisms could be identified in almost all RBDs and platelet disorders. It would be interesting to determine the heterozygosity frequencies of these polymorphisms.

There is a need for the formation of a consortium of Institutions working on the molecular pathologies of RBDs. The mutations and polymorphisms reported from scattered studies need to be incorporated into a National RBD registry. This will facilitate to offer quicker and cheaper diagnosis of the molecular pathologies and first trimester prenatal diagnosis in the Indian patients by screening for common mutations and the domains in the genes where most mutations are concentrated. In the absence of the information on molecular pathology of the index cases, we could offer second trimester prenatal diagnosis to families with FVII and FX deficiencies [13], [15]. In a family of GT who had one affected child, first trimester prenatal diagnosis using linkage assessment technique could be successfully given [23].

In disorders like FVII deficiency, the molecular pathology of the deficiency does not always correlate with the clinical manifestations of the patients [12]. Studies have shown that some of the polymorphisms in *F7* gene are associated with increased prothrombotic tendency. A670C transversion and a 37 base pair repeat polymorphism in intron 7 (seven or higher repeats) were shown to be independent risk factors for ischemic stroke in young adult patients [14]. These polymorphisms could protect the patients with severe FVII deficiency against bleeding manifestations. The present analysis along with other data from the world suggests that in many of the rare coagulation factor deficiencies there is a preponderance of mutations in specific exons. This exon preference has important implications in prenatal diagnosis where mutations in the family are not known.

In most RBDs, the mortality rate is not observed to be high. However, there is high degree of morbidity with heavy mucosal bleeds and menorrhagia in case of female patients. Patient families do tend to migrate to areas where accessibility to patient care is better. These migrations may give rise to founder mutations which are inherited to the progeny. However, not many founder mutations could be identified in the Indian patients with RBDs. One possible reason for this could be that in India, there are very few centers where molecular studies on coagulation proteins are being done. Other reason could be the premature death of these patients due to lack of adequate health infrastructure in this country or due to milder nature of some of these bleeding disorders they might not be visiting a haemostasis laboratory for further investigations. Moreover, these centers are located in extremely far away states from one another. For many patients with limited financial resources, it is not possible to travel these distances for proper diagnosis and treatment, hence there is a wide possibility that many of the patients with RBDs from remote regions of India are being missed. Though many new patients and mutations are being diagnosed, yet there is an increasing need for increased diagnostic and treatment facilities in India. The treatment products available currently do not cater to most rare factor deficiencies; moreover, they are very expensive and not easily accessible to most patients.

## Supporting Information

Checklist S1 **Shows the description and location of headings and subheadings in the manuscript.**
(DOC)

Flow Diagram S1 **Shows flow chart of the shortlisted published literature studied and analyzed for this review.**
(TIF)
