# Supplementary figures and images for: Molecular Pathology of Rare Bleeding Disorders (RBDs) in India: A Systematic Review
Source: PLoS One. 2014 Oct 2;9(10):e108683. doi: 10.1371/journal.pone.0108683 (PMC4183524; doi:10.1371/journal.pone.0108683)

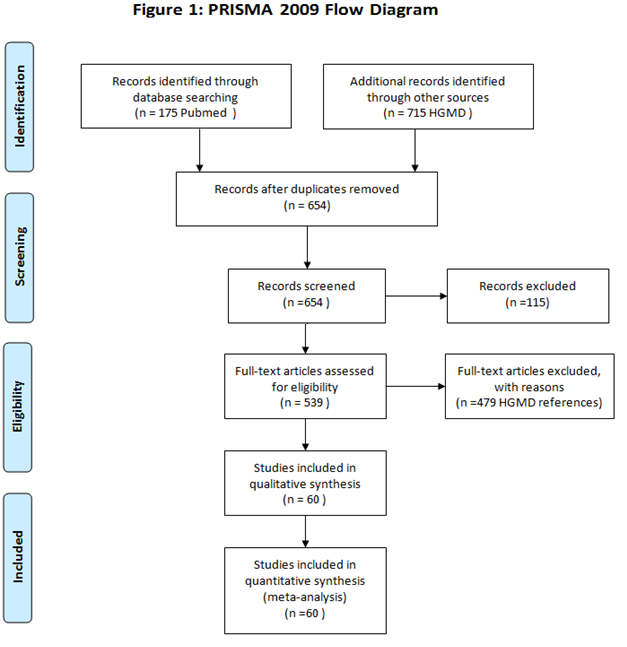

Supplement: Flow Diagram S1 — Shows flow chart of the shortlisted published literature studied and analyzed for this review. (TIF) [file pone.0108683.s002.tif]
